# Mutual Gaze: An Active Ingredient for Social Development in Toddlers with ASD: A Randomized Control Trial

**DOI:** 10.1007/s10803-020-04672-4

**Published:** 2020-09-07

**Authors:** Pamela Rosenthal Rollins, Adrienne De Froy, Michelle Campbell, Renee Thibodeau Hoffman

**Affiliations:** 1 Callier Center for Communication Disorders, University of Texas at Dallas, 1966 Inwood Road, Dallas, TX 75235 USA; 2 Pathways Early Autism Intervention, LLC, 255 Anglers Ridge, Bluff Dale, TX USA

**Keywords:** Autism spectrum disorder, ASD intervention, Mutual gaze, Social development

## Abstract

We examined the efficacy of an early autism intervention for use in early childhood intervention (ECI) and mutual gaze as a contributor to social development. Seventy-eight families were randomly assigned to one of three 12-week interventions: Pathways (with a mutual gaze component), communication, or services-as-usual (SAU). The Pathways/SAU comparison concerned the efficacy of Pathways for ECI, and the Pathways/communication comparison, mutual gaze. The Pathways group made significantly more change on social measures, communicative synchrony, and adaptive functioning compared with the SAU group and on social measures compared with the communication group. There were no group differences for communicative acts. The results support Pathways as a potential ECI program and mutual gaze as an active ingredient for social and communication development.

Autism spectrum disorder (ASD) is a complex, heterogeneous, neurodevelopmental disorder that severely compromises the development of social relatedness, reciprocity, social communication, joint attention, and learning. The Centers for Disease Control and Prevention estimated that 1 in 54 children are on the autism spectrum (Maenner et al. 2020). The continued rise in prevalence over the last 20 years has promoted substantial growth in research, advancing our understanding of the genetic, neurobiological, and developmental underpinnings of ASD (Mundy 2016; Zablotsky et al. 2019). There is now substantial evidence that the diminution of or deficits in social attention (i.e., social orienting, mutual gaze, and joint attention) are among the earliest behavioral indicators of ASD (Dawson et al. 1998; Jones and Klin 2013; Mundy 2016; Zwaigenbaum et al. 2005). This has led to improvements in early identification and recommendations for early intervention that meet the needs of toddlers with ASD (Schertz et al. 2012).

Early intervention can make a significant difference in a child’s joint attention, social communication, and adaptive functioning (Fuller and Kaiser 2019; Nahmias et al. 2019; Reichow 2012; Schertz et al. 2012). An accumulation of empirically supported autism interventions have investigated naturalistic developmental behavioral interventions (NDBIs) that focus on early-developing social and communicative outcomes for young children with ASD (Brian et al. 2016; Ingersoll and Gergans 2007; Kasari et al. 2015; Schreibman et al. 2015; Siller et al. 2013; Wetherby et al. 2018). NDBIs are rooted in social interactionist (Bruner 1981; Snow 1999; Vygotsky 1978) and transactional theories (Sameroff 2009) and use behavioral strategies, such as contingent natural reinforcers, to facilitate developmentally informed skills. The social-transactional approach describes the social and communication domains as usage-based systems, influenced by the continuous dynamic interactions of the child and the experience provided by the social environment (Sameroff 2009). Rather than focusing on direct teaching of isolated skills chosen by an adult, NDBIs focus on the toddler’s interacting with a communicative partner within the context of the toddler’s everyday environment. A core tenet of this approach is that the construction of the child’s social and communication systems is bidirectional and best achieved when the caregiver’s input is adapted to the child’s level of development within reciprocal interactions and is responsive to the toddler’s interests (Rowe and Snow 2019; Sameroff 2009).

When NDBIs are parent mediated, i.e., professionals work with parents to implement intervention strategies,^1^ they can be delivered in a manner consistent with the Individuals with Disabilities Education Act (IDEA) Part C Early Childhood Intervention (ECI) programs. Part C is a federal grant program that assists states in operating statewide community programs for infants and toddlers with disabilities. These programs necessitate the provision of family-centered and family capacity-building practices that enhance the family’s ability to promote the child’s development within the child’s authentic learning experiences (Adams and Tapia 2013; Division for Early Childhood [DEC] of the Council for Exceptional Children 2014; Schertz et al. 2011). In addition, the DEC recommends that instructional practices promote developmentally accessible outcomes while engaging the child in active learning (DEC 2014; Schertz et al. 2011).

Few research-based parent-mediated toddler programs meet Part C standards (Schertz et al. 2011; Wetherby et al. 2018), resulting in a disconnection between research-supported practices and implementation of these practices within community-based ECI programs (Siller et al. 2013; Wetherby et al. 2018). One way to bridge the research-to-practice divide is to address the principles of Part C within the research agenda and improve the match between the research-based intervention and the values, needs, skills, and resources of the community-based program (Horner et al. 2014; Schertz et al. 2011; Siller et al. 2013; Wetherby et al. 2018).

## Mutual Gaze as an Intervention Target

Many naturalistic and NDBI approaches to intervention for toddlers with ASD use strategies to work on social or communication skills that are within, or just above, the toddler’s level of development (Rollins 2016; Rollins et al. 2016; Ingersoll and Gergans 2007; Schertz et al. 2018; Wetherby et al. 2018). The advancement of research on typical infants and toddlers has provided new insights into developmentally appropriate instruction to promote social development (Rollins 2016; Schertz et al. 2018). In addition, we have a more refined understanding of the developmental unfolding and inter-relationships among mutual gaze, early dyadic social interaction, joint attention, and social communication in typical infants (Adamson et al. 2020; Rollins 2016; Camaioni 1993; Rowe and Snow 2019; Tomasello et al. 2005) and how a disruption in one or more of these areas can have cascading effects on later social and communication skills in children with ASD (Johnson et al. 2015; Jones and Klin 2013; Mundy 2016).

Early in infancy, mutual gaze is a significant aspect of dyadic interactions (Senju and Johnson 2009). Studies that use head cameras have found that infants’ everyday at-home visual experiences are dense with face input, allowing the infant to view both eyes (Fausey et al. 2016; Jayaraman et al. 2015). Around 2 months of age, with the onset of the social smile, there is an increase in mutual gaze that launches the dyad into a new quality of shared experiences (Rochat and Striano 1999; Stern 1985), at least in Western cultures. These dyadic face-to-face interactions reflect well-balanced, reciprocal exchanges of affect and emotions (Rochat and Striano 1999; Tomasello et al. 2005). This period of social cognition involves sharing emotions (Tomasello et al. 2005) and is the cognitive precursor to sharing attention/intention and social communication (Adamson and Russell 1999; Rochat and Striano 1999; Rollins and Greenwald 2013; Tomasello et al. 2005). Further, these dyadic social interactions are thought to facilitate recruitment of the social-brain network (Johnson et al. 2015).

Unlike in typically developing children, social attention to faces is diminished in children with ASD (Dawson et al. 1998; Dawson et al. 2004; Jones and Klin 2013; Zwaigenbaum et al. 2015). Using eye tracking technology, Jones and Klin (2013) found that infants with ASD exhibited a decline in eye gaze between 2 and 6 months, suggesting that they may miss out on opportunities to engage in social reciprocity. An intervention that focuses on mutual gaze may adjust the pattern of brain activity toward a more typical trajectory (Dawson et al. 2012; Johnson et al. 2015, Jones and Klin 2013; Senju and Johnson 2009).

The purpose of this study is twofold. The first is to examine the efficacy of Pathways parent-mediated NDBI for early autism that fits the guiding principles and service delivery model of IDEA Part C programs in low-resourced states, where the number of weekly visits by an interventionist may be limited. The second is to evaluate the mutual-gaze component of the Pathways intervention as an active ingredient for social development. The Pathways group was compared with two control groups: (1) a services-as-usual (SAU) group that received services from public and private community organizations; and (2) a parent-mediated NDBI that was identical to Pathways, except it used instructional strategies to facilitate social communication (i.e., communication intervention) instead of strategies to facilitate mutual gaze. Comparisons between the Pathways and SAU groups were used to understand the efficacy of Pathways as an ECI program, whereas comparisons between the Pathways and communication groups were used to understand the effects of the mutual-gaze component on developmental outcomes. Two research questions guided this study:

1. What are the effects of Pathways parent-mediated intervention on the development of social, communicative, and adaptive functioning skills when compared with the SAU group of ECI-aged toddlers with ASD?
2. What are the effects of Pathways parent-mediated intervention on the development of social, communicative, and adaptive functioning skills when compared with the communication intervention group of ECI-aged toddlers with ASD?

We hypothesize that a low-dosage, parent-mediated NDBI that coaches parents of toddlers with ASD to facilitate the early social phase of shared emotions may be an effective early intervention in low resourced states. Here we predict that toddlers in the Pathways group will make significantly more progress in social, communication, and adaptive functioning skills when compared to toddlers in the SAU group. Further, we hypothesize that engaging toddlers with ASD in face-to-face reciprocal interactions with mutual gaze will have cascading effects on development. Therefore, we predict that toddlers in the Pathways group will make more gains in social skills and similar gains in communication skills when compared with toddlers in the communication group whose parents coached them on strategies to facilitate communication, rather than mutual gaze.

## Methods

### Participants

Parents and toddlers aged 18–38 months were recruited through local infant toddler programs, community centers, advocacy groups, physicians’ offices, social media, and word of mouth. Eligibility criteria included: (a) having a chronological age of less than 38 months at the start of the study; (b) receiving an autism classification on the Autism Diagnostic Observation Schedule, Second Edition (ADOS-2; Lord et al. 2012), administered by an ADOS-2 reliable examiner; (c) having no other medical, neurological, or genetic concerns or disorders; and (d) having a primary home language of English or Spanish.

**Fig. 1:**
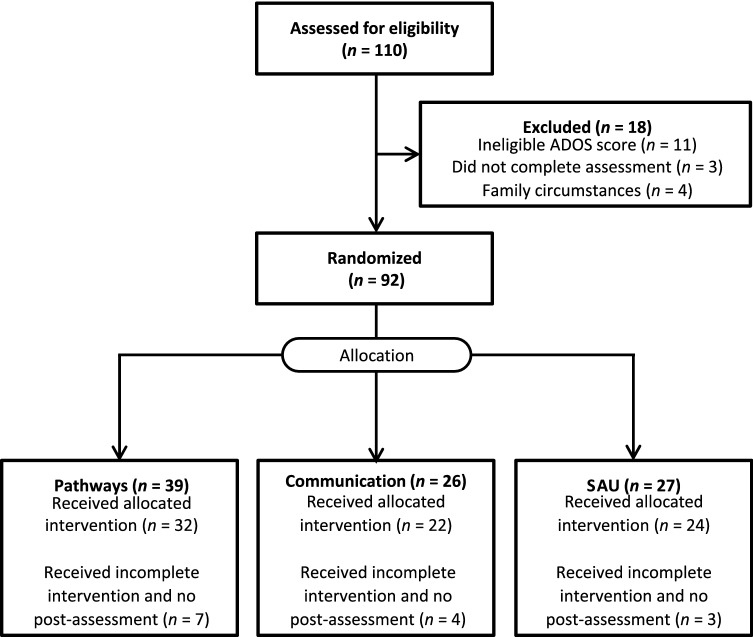
Diagram of study flow (Rollins et al. 2019)

Among the 110 toddlers screened, 92 met the inclusion criteria and were enrolled in the study. The families were randomly assigned into the Pathways (*n* = 39), communication (*n* = 26), and SAU control (*n* = 27) groups (Fig. 1). Fourteen families did not complete the intervention or receive post-assessment because they had a prolonged illness, moved out of the area/country, or an intensive service opportunity became available to them, and they could not be reached to schedule post-intervention assessments. The remaining 78 children received the full intervention and post-assessments. There were no significant differences in the background variables between the families who did not complete the intervention and post-assessment procedures from those who did (Table 1); however, age in months, nonverbal IQ, and parenting stress have small effect sizes.

**Table 1:** Means, standard deviations, and t-statistics for group equivalencies on baseline characteristics of participants who did not complete (DNC) and completed the intervention

Characteristic	DNC (*n* = 14)	Completed (*n* = 78)	Independent-samples *t*-test		
	*M* (*SD*)	*M* (*SD*)	*t* (*df*)	*p* value	*d*
Age in months	27.2 (3.9)	28.2 (5.2)	.72 (91)	.47	.22
ADOS-2 severity	8.6 (2.2)	8.4 (1.7)	− .42 (90)	.67	.10
NVIQ	67.1 (22.4)	63.3 (16.0)	− .63 (17)	.54	.20
VIQ	40.1 (21.7)	43.1 (22.2)	.48 (91)	.63	.14
VABS communication	66.4 (18.8)	66.9 (14.2)	.12 (91)	.90	.03
VABS social	73.6 (9.7)	74.1 (9.0)	.20 (91)	.84	.05
Mother’s education	14.2 (3.6)	14.6 (2.7)	.43 (91)	.67	.13
Total stress percentage	75.5 (11.8)	72.1 (18.8)	− .68 (91)	.50	.25

ADOS-2, Autism Diagnostic Observation Schedule, Second Edition; NVIQ, nonverbal IQ; VIQ, verbal IQ; VABS, Vineland Adaptive Behavioral Scale composite score

NVIQ, VIQ, and VABS scores are based on *M* = 100, *SD* = 15. Total Stress scale from the Parenting Stress Index, Fourth Edition-Short Form (85–89 = high stress and 90–100 = clinically stressed). *d* = Cohen’s *d* measure of effect size, where *d* = .2 is a small effect; .5, medium effect; and .8, large effect

Descriptions of the 78 children who completed the post-intervention assessments are presented in Table 2. The constitution of the sample mirrored the statewide ECI estimates (Texas Health and Human Services, n.d.). Toddlers, on average, had significant cognitive challenges and came from culturally and socioeconomically diverse families. The sample had a low percentage of Caucasian families and a high percentage of Children’s Health Insurance Program (CHIP) Medicaid-eligible families.

**Table 2:** Means, standard deviations, and ANOVAs for group equivalencies on continuous baseline characteristics and frequencies, and Chi square results for group equivalencies on categorical demographics across conditions

Characteristic	Pathways (*n* = 32)		Communication (*n* = 22)		SAU (*n* = 24)		Group comparisons	
	*M*	(*SD*)	*M*	(*SD*)	*M*	(*SD*)	*F*(2, 75)	*ɷ*^*2*^
Age in months	28.8	(4.7)	29.3	(5.2)	26.4	(5.5)	2.32	.03
ADOS-2 CSS	8.4	(1.8)	8.3	(1.9)	8.60	(1.5)	0.25	.02
MSEL	54.9	(10.1)	58.7	(12.5)	55.2	(8.9)	0.96	− .00
Mother’s education (years)	14.2	(2.6)	14.9	(2.7)	14.8	(2.9)	0.71	− .01
VABS social	73.4	(7.4)	73.8	(9.0)	74.9	(10.8)	0.19	− .02
VABS adapt	74.0	(8.4)	74.9	(11.0)	73.6	(9.9)	0.12	− .02
Total stress percentage	71.0	(21.9)	73.5	(17.3)	72.2	(16.3)	0.12	− .02
Social eye gaze	5.4	(8.4)	5.1	(5.2)	7.8	(10.6)	0.75	− .01
Comm synchrony	3.3	(4.9)	3.2	(6.3)	5.2	(9.5)	0.66	− .01
Total CAs	7.2	(10.5)	9.6	(19.9)	10.0	(15.9)	0.27	− .02
Pragmatic flexibility	3.9	(4.8)	4.5	(6.3)	3.7	(3.9)	0.19	− .02

	%	%	%	χ^2^ (df)	*V*
Male	69	86	79	2.38(2)	.18
Ethnicity				1.72(4)	.11
Caucasian	22	28	21		
Hispanic	50	45	38		
Other	28	27	42		
Income				4.95(6)	.18
< $25,000	19	32	29		
$25,001–$50,000	34	27	17		
$50,001–$100,000	19	27	21		
$100,001+	28	14	33		
CHIP/Medicaid eligible	47	46	46	0.01(2)	.01

ADOS-2 CSS, Autism Diagnostic Observation Schedule, Second Edition, Total Calibrated Severity Score; MSEL, Mullen Scales of Early Learning, Early Learning Composite score; VABS Social 1 and VABS Adapt 1, Vineland Adaptive Behavior Scale, Second Edition Socialization domain score, and Adaptive Behavior Composite, respectively

MSEL and VABS scores are standard scores based on *M* = 100, *SD* = 15; Comm Synchrony = Synchrony of Communicative Behaviors; Total CAs = Total communicative acts; CHIP = Children’s Health Insurance Program; *ɷ*^*2*^ = omega squared, effect size measure for which .01 = small effect and .06 = medium effect; *V* = Cramer’s V, effect size measure for which .1 = small effect and .3 = medium effect

*p < .05

At baseline, prior to the 12-week intervention phase, a clinical researcher asked the parent to report the number of hours that the toddler received which type of community-based services. The mean number of hours of community-based intervention received prior to the start of the study was 1.38 h/week, (*SD* = 0.95, range = 0–3.13) for the Pathways group; 1.05 h/week (*SD* = 0.69, range = 0–2.50) for the communication group; and 1.92 h/week (*SD* = 2.17, range = 0–9) for the SAU group. A one-way analysis of variance (ANOVA) revealed no significant differences in hours of intervention prior to the start of the study (*F*(2,73) = 2.5, *p* = .09, *ɷ*^*2*^ = –0.04). Although the majority of toddlers in all groups were receiving ECI services, proportionately more children who were later randomized into the Pathways group received no intervention before the study (Table 3). All parents agreed to participate in the study, using an informed consent procedure approved by the university’s Institutional Review Board. There was no cost for participating in the study.

**Table 3:** Means and standard deviations for parent-reported hours per week of intervention by type that toddlers received prior to the study across treatment group

Intervention Type	Pathways		Communication		SAU	
	*n*	*M* (*SD*)	*n*	*M* (*SD*)	*n*	*M* (*SD*)
ECI	18	1.43 (0.68)	16	1.25 (0.60)	17	1.26 (0.60)
Private/community services	4	1.25 (0.50)	3	1.00 (0.00)	5	4.30 (3.86)
No intervention	8		3		2	

Data are missing from two children in the social group and one child in the communication group

### Study Design

This study used a randomized waitlist control design. The principal investigator used a computer-generated list of random numbers to allocate participants to treatment conditions. Sealed envelopes were used for allocation concealment and were opened after all baseline assessments were completed. Clinical researchers administered the assessments and provided the intervention for the Pathways and communication groups. Recruiting, intake, and pre- and post-intervention testing procedures were identical for the three conditions. The Pathways and communication groups received 12 weeks of project-related home visits. The SAU control group received 12 weeks of intervention from community early-intervention providers. Families in the SAU control group could elect to receive the Pathways intervention at no charge when they completed the study.

### Clinical Researcher Qualifications and Training

Four clinical researchers participated in the study. They were responsible for administering the assessments and providing the intervention. Three had a master’s degree and were certified speech-language pathologists. The fourth had a bachelor’s degree in education and 15 years of experience as an early interventionist. Two of the speech pathologists were bilingual (English–Spanish) and, when requested, provided assessments and interventions in Spanish.

Prior to the start of the study, the clinical researchers achieved reliability on the ADOS-2 (Lord et al. 2012) with one of the authors, who is an ADOS-2-reliable examiner. Specially, each research clinician independently coded and scored video-recorded and live administrations of the ADOS-2, then checked point-by-point reliability until 90% inter-examiner agreement was achieved with the ADOS-2-reliable examiner. This was repeated until 90% reliability was achieved on three consecutive ADOS-2 administrations, which could be video recorded or live. Taking into account the age and ability level of the children in the study, each research clinician demonstrated standardized administrations on three separate occasions for the Toddler Module and Module 1 only. During the study, two clinical researchers were present for each assessment. One administered the ADOS-2 while the second assisted. The two researchers scored the assessments together and discussed any issues that might have come to light. Finally, each researcher clinician independently coded three separate ADOS-2s, every 3 months, to check that there was 80% reliability among the group, which was the case in each instance.

A similar procedure was followed to obtain reliability on other standardized assessments. Specifically, the clinical researchers reviewed administration and scoring procedures and subsequently practiced administering and scoring the test with non-project children and adults until there was 90% reliability with one of the authors. Two clinical researchers were present for child assessments (one administered while the second assisted). Only one clinical researcher administered the adult standardized interviews. All tests were independently scored by two people to check accuracy.

With regard to intervention, the clinical researchers were trained to fidelity, using practice children prior to the start of the study. In addition, they participated in weekly supervision with the first author where they reviewed video recordings of treatment sessions.

### Intervention Procedures

The Pathways intervention (Campbell and Hoffman 2012) was developed by practitioners in a community-based Part C program. The Pathways and communication interventions are targeted, manualized programs with English and Spanish versions of manuals in both print and digitized audio formats. Parents received family-centered coaching for 1.5 h/week, using multi-modal strategies to enhance the family’s capacity to promote their child’s development within the natural environment (Table 4). Each unit of the intervention introduced information about ASD and explicit instructions on two or three interactional strategies (Table 5). The interventions utilized play activities and daily routines appropriate to a family’s culture and encouraged parents to use the interactional strategies throughout much of the child’s day.

**Table 4:** Sequence of session and coaching strategies used with project-related treatment groups

Activity	Time (mins)	Description of coaching strategy
Introduction	10	Relationship building. Interventionist and parent review and discuss activity plan, progress, and barriers to learning from the previous week
Observation	10	Interventionist collects a digitized video, observing the parent implementing previously learned intervention strategies
Reflection and evaluation	10	Interventionist reviews video with parent. Together, they identify strategies that are being used effectively and those that are challenging for the parent. Interventionist and parent review the self-assessment rating together, reflecting on and identifying their comfort level, successes, and challenges with each strategy
Demonstration	15–20	Interventionist and parent discuss how implementation strategies can be improved, with opportunities for the parent to ask questions. Interventionist demonstrates strategies with the toddler, providing a verbal narrative. Parent practices strategies and is given feedback
New material	10–15	New print material from the manual is presented to the parent only if the parent is ready to move to the next unit. Interventionist verbally reviews new material, which includes explicit information about each strategy
Demonstration	10–15	Interventionist demonstrates intervention strategies with child, narrating what he or she is doing, while parent observes
Parent practice	10–15	Parent takes the lead practicing strategies with the child. Interventionist provides positive feedback and suggestions for improvement. Interventionist engages parent in problem solving and reflection about parent’s implementation of the new strategies during practice
Develop activity plan	10	Parent and interventionist plan how strategies can be embedded in activities and routines during the upcoming week and create an activity plan. Interventionist addresses any final questions and concerns that parent may have

**Table 5:** Interactional strategies for each unit across project-related treatment groups

Unit	Pathways	Communication
1	**Setting up the environment I** Follow child’s lead Limit talking and demands Use wait time	**Setting up the environment I** Follow child’s lead Limit talking and demands Use wait time
2	**Setting up the environment II** Limit distractions Organize toys Use face-to-face positioning Join in and play Engage in social sensory, family, and daily routines	**Setting up the environment II** Limit distractions Organize toys Use face-to-face positioning Join in and play Engage in social sensory, family, and daily routines
3	**Facilitate mutual gazing**^**a**^ ABCs of behavior Contingent natural reinforcement Practice new skills in different activities and environments and with different people	**Facilitate communication**^**b**^ ABCs of behavior Contingent natural reinforcement Practice new skills in different activities and environments and with different people
4	**Use of animation** Exaggerate gestures, facial expressions, and voice quality	**Use of animation** Exaggerate gestures, facial expressions, and voice quality
5	**Encourage Imitation** Imitate toddler’s vocalizations and actions Put it all together in daily routines	**Encourage Imitation** Imitate toddler’s vocalizations and actions Put it all together in daily routines
6	**Balancing Interaction** Add something new to the interaction Create opportunities for reciprocal imitation	**Balancing Interaction** Add something new to the interaction Create opportunities for reciprocal imitation

*ABCs of behavior* antecedent, behavior, and consequences

^a^Mutual gaze strategy: Engage toddler in mutual gaze during motivating face-to-face routines, without verbal, visual, or physical prompts, followed by contingent natural reinforcement

^b^Communication strategies: Create situations for communication during motivating routines and activities of daily living; use a core vocabulary, modeling, and prompting communicative attempts; follow toddler’s communication by contingent natural reinforcement

An important feature of the intervention is that the units are cumulative. Each unit builds on the preceding units to slowly move the parents toward increasingly more sophisticated levels of interaction with their toddler. Once an intervention strategy was introduced, the parent was expected to use that strategy for the remainder of the study. Each week, parents rated their perceived level of competence on each interactional strategy from the current unit and all previous units. Parents had the opportunity to discuss where they felt they were successful and where they felt they had challenges. A new unit was introduced when the parent was comfortable with applying all current and previous learned strategies, and the interventionists rated the parent as implementing the strategies with fidelity, using a 4-point Likert scale (Rollins et al. 2019). Consequently, some units were presented only one time, whereas other units were presented over two or more sessions, according to the parent’s abilities. Overall, the parents’ fidelity ranged from 86 to 100% (*M* = 96%, *SD* = .04).

### Differences Between the Pathways and Communication Intervention

Both the Pathways and the communication interventions promoted dyadic face-to-face reciprocal interactions within social sensory and daily routines. The difference between the two interventions were the strategies presented in Unit 3 (Table 5). Parents in the Pathways group were coached on strategies to engage their toddler in mutual gaze during motivating face-to-face activities and routines, followed by contingent natural reinforcement. The strategy requires the parent to actively establish eye gaze without verbal, visual, or physical prompts when the toddler was not spontaneously engaged in mutual gaze. In contrast, parents in the communication group were coached on strategies to facilitate communication. Strategies included creating situations for communication during motivating routines and activities of daily living, using a core vocabulary, modeling, and prompting communicative attempts. The toddler’s communication was followed by contingent natural reinforcement. Parents in both of the groups were introduced to Unit 3 between Weeks 3 and 5 of the study (*M* = 4.04 weeks, *SD* = .64). Consequently, although the difference between the two interventions was only the strategies taught in Unit 3, parents implemented the Unit 3 strategies with their children for a period of seven to nine weeks.

### Amount of Project and Non-project Intervention

Families in the Pathways and communication groups had to suspend other speech, developmental, or applied behavior analysis (ABA) services for the duration of the study. These families received only 1.5 h/week of project-related intervention for 12 weeks. The SAU control group received intervention from community early-intervention providers during the 12-week intervention period. A clinical researcher asked the parents in the SAU group to report the number of hours that their child attended (a) early intervention, (b) speech/ language services, (c) ABA services, and (d) other community-based services during the post-intervention assessment. Parents reported that the SAU group received an average of 6.65 h (*SD* = 8.90) of therapy per week, with a range of 1–28 h/week. Of the 24 children, 13 were enrolled in a state-sponsored, local ECI program and received all of their services from that agency (*M* = 1.69, *SD* = .79 h/week). Six children had their state services supplemented with speech language and ABA services (*M* = 15.58, *SD* = 10.74 h/week). Finally, five children received all of their services privately or as part of other community-based services (*M* = 7.9, *SD* = 9.88 h/week).

### Video Data Collection and Coding Procedures

The clinical researchers collected video recordings of parent-child interactions in the families’ homes at baseline, prior to randomization, and at post-intervention. All parent-child dyads were digitally recorded for 10 min, using an iPad 2 for a wide-angle recording of the interaction (Video Stream 1) and with hidden-camera eyeglasses worn by the parent to capture the child’s eye contact (Video Stream 2). Parents were instructed to play with their child as they typically do. The interventionists gave the parents instructions on where to place the hidden-camera glasses to assist with data collection but made no suggestions or recommendations about the interactional strategies.

For each recording, the two streams of digitized videos (i.e., iPad and glasses) were segmented into 2-second intervals and time-linked, using the conventions of the Child Language Data Exchange System (CHILDES; MacWhinney 2000). This allowed for data reduction, using both partial interval coding (Yoder and Symons 2010) and CHILDES transcription and coding methodology (MacWhinney 2000). Transcription was conducted at the level of the utterance and included all verbal, vocal, and gestural behaviors bounded by a pause or change in a conversational turn (Pan et al. 2005).

For each recording, each measure (defined below) was coded by a different team of research assistants during separate passes through the transcript. All coders received a multimedia coding manual with definitions and examples of coding categories. Coders trained on practice videos until they achieved substantial inter-rater agreement, measured by obtaining a Cohen’s kappa coefficient of .80 or above. To address coder drift, all coders attended weekly lab meetings to discuss coding, and their reliability was checked every 3 months. Most coders maintained good reliability throughout the study. When coder drift was identified, the unreliable coder was retrained on practice videos until a kappa coefficient of .80 was achieved. In addition, a master coder reviewed the unreliable coder’s files from the previous 3 months and recoded when necessary. All coders were blind to group assignment and time (i.e., pre- or post-intervention).

### Measures

Standardized and video assessments were conducted at baseline prior to randomization and post-intervention, within 2 weeks of the start and stop of the intervention phase. The clinical researchers were blind to group assignment pre-intervention but not post-intervention. A clinical researcher who did not coach the target family conducted post-intervention assessments. All assessments were administered in the family’s home or at a convenient location.

#### Video-Coded Measures

Social eye gaze, number and diversity of communicative acts, and synchrony of communicative behaviors were extracted from the 10-min coded transcript files, using the utilities of the Computerized Language Analysis software package (MacWhinney 2000). A final inter-rater reliability assessment was conducted on each measure after project-related coding was completed. Specifically, a second rater independently coded 20% of the baseline videos, chosen at random, and 20% from post-intervention videos, chosen at random, for each video-coded measure. Inter-rater reliability was estimated using a Cohen’s kappa statistic, which takes into account chance agreement. Kappa statistics between 0.80 and 0.90 are considered substantial to near-perfect agreement (Landis and Koch 1977).

*Social eye gaze* was used to measure the amount of time that the toddler initiated eye gaze that was paired with positive affect. Social eye gaze was an interval-coding measure determined from the hidden-camera eyeglasses video stream. The measure was defined as the number of 2-second intervals that the toddler initiated social eye gaze by turning his or her head to look in the parent’s eyes, coupled with a smile. The mean kappa coefficient for social eye gaze was .89 (*SD* = .14).

*Number and diversity of communicative acts (CA)* were used to measure communicative ability. They were derived from the transcription and coding measures. CAs were identified, using Wetherby and Prizant’s (2002) definition of an interactive behavior, as consisting of “gesture, vocalization, or verbalization that is directed towards an adult and serves a communicative function” (p. 47) and coded for intention, using the Inventory of Communicative Acts-Abridged (INCA-A; Ninio et al. 1994). The INCA-A is based on speech-act theory (Austin 1962; Searle 1976) and on studies of events in face-to-face interaction (Goffman 1974; Streeck 1980) that emphasize the importance of socially constructed communicative interactions. The system identifies and codes communicative intent at the level of the social interaction (i.e., regulating another’s behavior, participation in a routine, and discussion around a joint focus of attention) and at the level of the utterance, thus acknowledging the existence of an organization of talk at a level higher than the single utterance (Dore and McDermott 1982; Streeck 1980). The number of CAs captured the frequency of communication within 10 min, while the diversity of CAs captured the number of different interaction-speech act combinations used in 10 min (Snow et al. 1996). The mean kappa coefficient for transcription was .80 (*SD* = .10) and .81 (*SD* = .19) for diversity of communicative acts.

*Synchrony of communicative behaviors (SCB)* was used to measure the social sophistication of the communicative behavior. It was defined as the number of communicative acts in 10 min that had two or three temporally overlapping behaviors. Communicative behaviors included words, vocalizations, gestures, and social eye gaze. Communicative behaviors produced in synchrony are developmentally and socially more sophisticated than are communicative behaviors produced in isolation (Heymann et al. 2018). The mean kappa coefficient for SCB was 0.99 (*SD* = 0.03).

#### ASD Classification

The ADOS-2 (Lord et al. 2012) was used to confirm a research diagnosis of ASD at intake prior to randomization. The ADOS-2 is a semi-structured evaluation of communication, social interaction, play, and restricted/repetitive behaviors for children who are suspected of having ASD. The ADOS-2 provides scores related to a child’s Social Affect (SA), Restricted and Repetitive Behavior (RRB), and Overall Total score. For the present study, The ADOS-2 Toddler Module and Module 1 were administered by a research clinician who was trained on site to be ADOS-2 reliable (see Qualifications and Training above). The internal consistency reliability for these two modules is high for the SA domain (.87–.92) and adequate for the RRB domain (.50–.66; McCrimmon and Rostad 2014). The Toddler Module, which is intended for toddlers 12–30 months of age, was administered to 29, 14, and 19 toddlers, later randomized into the Pathways, communication, and SAU groups, respectively. Module 1 of the ADOS-2, which is intended for children aged 31 months and older whose language abilities range from no speech to simple phrases, was administered to the three, eight, and five toddlers, randomized into the Pathways, communication, and SAU groups, respectively. All ADOS-2 scores were converted to a Calibrated Severity Score (CSS) to allow comparisons across modules (Esler et al. 2015; Gotham et al. 2009).

#### Developmental Functioning

The Mullen Scales of Early Learning (MSEL; Mullen 1995) was used to estimate overall developmental functioning at baseline prior to randomization. The MSEL was administered by the research clinicians (see Qualifications and Training above). The MSEL is a standardized, direct assessment of development for young children (ages 0–68 months) that yields age-equivalency scores for gross and fine motor skills, visual reception, and receptive and expressive language. The MSEL yields the Early Learning Composite (ELC), a single standardized score (mean = 100, *SD* = 15), to measure overall developmental functioning. MSEL has good test-retest reliability (.80–.70, depending on interval between testing) and high internal consistency (.80–.75, depending on the subscale). The ELC may be used as an as overall measure of developmental functioning (Bishop et al. 2011).

#### Social and Adaptive Functioning

The parent interview form of the Vineland Adaptive Behavior Scales, Second Edition (VABS-II; Sparrow et al. 2005) was administered by the research clinicians (see Qualifications and Training above) at baseline prior to randomization and at post-intervention to measure social and adaptive functioning skills. The VABS-II is a standardized test of adaptive functioning for individuals from birth to age 90 years. The test yields an adaptive behavior composite score and domain scores for communication, daily living, socialization, and motor development and has good test-retest reliability (.88–.92).

#### Parenting Stress

The Parenting Stress Index, Fourth Edition, Short Form (PSI-4-SF; Abidin 2012) is used to assess total parental stress (Total Stress), which is an indicator of risk for dysfunction. The PSI-4-SF was administered by the research clinicians (see Qualifications and Training above) at baseline prior to randomization and at post-intervention to measure parenting stress. The PSI-4-SF is a standardized parent questionnaire on which parents rate agreement on 36 items, using a 5-point Likert scale. Total Stress is expressed as percentile scores, for which higher scores indicate higher levels of stress. The PSI manual reports good test-retest reliability for the total score (.96) and good internal consistency (.98).

#### Change (∆) Measures

The change from pre- to-post-intervention was used to create outcome variables for social eye gaze, VABS social and adaptive functioning scores, number and diversity of communicative acts, and synchrony of communicative behaviors. (Table 6). Changes were calculated as a difference score (post-intervention minus baseline) on each measure. A participant’s score of 0 indicates that the participant’s score post-intervention was the same as the participant’s score at baseline. Conversely, positive values indicate that a participant’s scores were higher at post-intervention than at baseline, and a negative value indicates that a participant’s scores were lower at post-intervention than at baseline. Difference scores are an appropriate measure to estimate change because they have less bias than do residual change scores in the presence of randomization and floor effects (Jennings and Cribbie 2016). In addition, group equivalency at baseline (see below) and randomization provides protection from the regression-toward-the-mean effects (Allison 1990).

**Table 6:** Means and standard deviations for change (post-intervention minus baseline) outcome measures across treatment groups and effect size comparisons

Characteristic	Pathways (*n* = 32)			SAU (*n* = 24)			Communication (*n* = 22)			Pretest-posttest *d*	
	*M*	(*SD*)	Range	*M*	(*SD*)	Range	*M*	(*SD*)	Range	P/S	P/C
ΔSocial Eye Gaze	21.41	(21.63)	− 5, 76	− 0.74	(8.92)	− 20, 20	4.23	(7.57)	− 6, 23	2.32	2.47
ΔVABS Social	14.84	(10.69)	− 2, 38	4.48	(8.70)	− 15, 18	8.18	(8.73)	− 7, 24	0.88	0.54
ΔComm	0.12	(1.10)	− 2, 3	− 0.33	(0.75)	− 2, 2	0.17	(1.04)	− 3, 2	0.18	0.05
ΔSCB	8.47	(10.73)	− 3, 44	0.57	(5.15)	− 11, 14	7.05	(11.44)	− 12, 38	1.04	0.25
ΔVABS Adapt	44.56	(23.89)	− 2, 95	20.91	(18.08)	− 4, 55	32.41	(24.02)	− 11, 86	0.56	0.26

Baseline and post-intervention assessments were conducted within 2 weeks of the start and stop the intervention phase; ΔVABS Social and ΔVABS Adapt are calculated on the sum of raw scores; ΔComm = change in composite of number and diversity of communicative acts; ΔSCB = change in synchrony of communicative behaviors; P/S= comparison between the Pathways and SAU groups; P/C= comparison between the Pathways and communication groups. *d* = standardized mean difference, a measure of effect size for pre-test/post-test designs that uses the standard deviation of the baseline scores on each measure instead of the standard deviation of the change score (Feingold 2013); *d* = .2 is a small effect; .5, medium effect; and .8, large effect

The VABS social and adaptive functioning change scores were calculated on the sum of raw scores because standardized scores would compare some (but not all) children to a different reference group at baseline and post-intervention. Finally, changes in the number and diversity of CAs were highly correlated (*r* = .79, *p* < .0001). Change in communicative intentions by interchange category revealed that the majority of communicative interactions were used to regulate another’s behavior (*∆* Behavior Regulation), reducing the variability in the diversity measure (Table 7). To retain the variation from both measures, we combined change in number and diversity of communicative acts into a single change in the communication variable, using principal components analyses. The resulting composite (*∆* Comm) accounted for 89% of the variance in the original two variables.

**Table 7:** Minimum, maximum and quartiles for change (post-intervention minus baseline) in communicative intention measures across treatment groups

Communicative intention	Minimum	Q1	Median	Q3	Maximum
ΔBehavior regulation					
Pathways	− 15.0	1.0	4.5	11.5	49.0
Communication	− 19.0	0.0	3.5	13.3	38.0
SAU	− 24.0	− 1.0	0.5	8.8	27.0
ΔRoutine					
Pathways	− 3.0	0.0	0.5	7.0	31.0
Communication	− 19.0	0.0	0.0	9.0	46.0
SAU	− 14.0	0.0	0.0	1.0	16.0
ΔMutual attention					
Pathways	− 6.0	0.0	0.0	1.8	31.0
Communication	− 3.0	0.0	0.0	1.0	10.0
SAU	− 1.0	0.0	0.0	0.0	21.0

*Q1* 25th percentile, *Q3* 75th percentile

### Power Analysis

G-Power (Faul et al. 2009) was used to calculate the sample size necessary to find a medium effect (*f*^*2*^ = .25) for *α* = .05 and power (1-β = .80) when conducting *R*^*2*^-increment testing in a fixed model linear regression with one test predictor and between two and 14 total predictors. Effect size estimates were based on the author’s previous studies (Rollins 2016; Rollins et al. 2016), which used similar measures to those proposed here. The results indicated that a sample size of 55 participants is required to find a medium effect with a .2 probability of failing to detect a genuine effect.

### Data Analytic Strategy

IBM SPSS statistical package version 26 was used to analyze the data. Preliminary analyses were conducted to establish pre-intervention group equivalencies on all baseline characteristics found in Table 2 and to identify potential covariates for subsequent regression models. One-way ANOVAs were used to test group equivalencies for the continuous measures, and Chi square analyses were used for the categorical variables. To identify potential covariates, we performed Pearson product correlations between continuous baseline variables and outcome measures and one-way ANOVAs between categorical baseline variables and outcome measures. Covariate analyses were performed separately for the Pathways/SAU group comparison and for the Pathways/communication comparison. Following the principle of parsimony, baseline variables were retained as covariate control variables in subsequent regression models only when they were found to relate to an outcome variable of interest.

To address the research questions, we conducted a series of regression analyses for each question, using *group* as a dummy-coded variable. The first set of analyses compared the Pathways and the SAU groups, and the second set concerned the Pathways/communication group comparison. For each regression model, covariates, if applicable, were entered into the model first, followed by the dummy-coded group variable. Consequently, the unstandardized regression coefficient (*b*_1_) for the dummy-coded group variable estimates the mean difference between the change (adjusted for covariates when applicable), from pre-to post- intervention, for the Pathways and the respective control group (SAU or Communication). A positive coefficient indicates that the mean change for the Pathways group is *b*_1_ units of measurement more than the mean change for the respective control group. Treatment effects were assessed by the magnitude of the effect obtained from the coefficient of determination (i.e., *R*^*2*^) when only the dummy-coded group variable was in the model or the change in *R*^*2*^ (i.e., Δ*R*^*2*^) from the base model that contained the covariates to the full model that contained the covariates and the dummy-coded group variable. The specific effect size calculation was *f*^2^ = *R*^*2*^/1 − *R*^*2*^, and interpretation was based on Cohen (1992), whereby *f*^2^ = effect size, for which ≥ .02 suggests a small effect; ≥ .15, a medium effect; and ≥ .35, a large effect. In addition, the unadjusted treatment effects are reported as the standardized mean difference in change scores between two groups, or *d* (Table 6). Following Feingold’s (2013) recommendations for calculating *d* for pre-test/post-test designs, the standard deviation of the baseline scores, rather than that of the change score, on each measure was used in the calculation. Interpretation of *d* pre-test/post-test designs also is based on Cohen (1992), whereby *d* = .2 suggests a small effect; *d* = .5, a medium effect, and *d* = .8, a large effect. Further, when covariates were present, we tested for their interaction with group assignment to identify predictors of treatment. All assumptions of regression were analyzed, and no model violations were present.

## Results

### Preliminary Analyses

Pre-treatment group equivalence was analyzed using one-way ANOVAs for continuous variables and Chi square analyses for categorical variables. There were no group differences in baseline variables, and the effect sizes for these comparisons ranged from miniscule to small (Table 2). Pearson product correlations were used to identify continuous covariates for subsequent analyses. For the Pathways and SAU group comparison (*n* = 56), Δ Comm was related to baseline VABS Social (*r* = .31, *p* = .022) and baseline VABS Adapt (*r* = .44, *p* = .001); ΔSCB also was related to baseline VABS Social (*r* = .30, *p* = .023) and baseline VABS Adapt (*r* = .38, *p* = .004); and ΔVABS Adapt was related to age (*r* = .34, *p* = .011), MSEL (*r* = .48, *p* = .0001), and baseline VABS Adapt (*r* = .42, *p* = .001). For the Pathways and communication group comparison (*n* = 54), ΔSocial Eye Gaze was related to baseline MSEL (*r* = − .28, *p* = .038), ΔComm was related to baseline VABS Adapt (*r* =.29, *p* = .031), and ΔVABS adapt was related to baseline MSEL (*r* = .32, *p* = .018). One-way ANOVAs found no significant categorical covariates for either the Pathways/SAU group comparison or for the Pathways/communication group comparison.

### Pathways and SAU Group Comparison

The regression coefficients on the dummy-coded group variable and the associated partial *F*-statistic and effect size estimates for the outcome variables are presented in Table 8. Any covariates that were entered into the model before the group variable are listed in the first column of the table. When covariates were entered in the model, as they are for ΔComm, ΔSCB, and ΔVABS adapt, the regression coefficients reflect the adjusted mean difference in change on the outcome measures. The results suggest that the difference in change from baseline to post-intervention was significantly greater in the Pathways group for social eye gaze and VABS Social, the two social measures. The magnitude of the effect for both measures is large. The large effect (*f*^2^) obtained from the regression models associated with these two measures also are reflected in the *d* statistics reported in Table 6.

**Table 8:** Unstandardized regression coefficients and confidence intervals for the dummy-coded group variable, adjusted for covariates, and the associated partial F-statistic and effect size estimates for the comparison of the Pathways intervention and SAU

Covariate	*b*_Group_	95% CI for *b*_Group_		*SE*	*Partial F*	*df*	*p* value	R^2^/ΔR^2^	*f*^2^
		LL	UL						
			ΔSocial Eye Gaze						
None	22.03	12.64	31.43	4.70	22.01	1, 54	.0001	.29	.41
			ΔVABS Social						
None	10.76	5.40	16.12	2.67	16.20	1, 54	.0001	.23	.30
			ΔComm						
VABS adapt, VABS social	0.24	− 0.27	0.75	0.25	0.92	1, 51	.341	.01	.01
			ΔSCB						
VABS adapt, VABS social	6.48	1.70	11.27	2.38	7.37	1, 52	.009	.11	.12
			ΔVABS adapt						
Age, MSEL, VABS adapt	21.54	11.59	31.49	4.95	18.90	1, 51	.0001	.18	.22

*f*^2^ = effect size, where ≥.02 = small effect, ≥.15 = medium effect, and ≥.35 = large effect

*b*, unstandardized beta; CI, confidence interval; LL, lower limit of confidence interval; UL, upper limit of confidence interval; VABS Social and VABS Adapt, sum of raw scores of the Vineland Adaptive Behavior Scale, Second Edition Socialization domain and Adaptive Behavior Composite; Comm, communication, a measure that composites the number and diversity of communicative acts; SCB, synchrony of communicative behaviors; MSEL, Mullen Scales of Early Learning, Early Learning Composite score

The difference in change on the communication composite, adjusted for baseline VABS Adapt and VABS Social, was not significant, and the effect was miniscule, as it was in the unadjusted effect size reported in Table 6. Together, the two covariates accounted for 21% of the variation in the ΔComm (*R*^*2*^ = .21, *f*(2,52) = 6.85, *p* = .002). In contrast, the difference in change for the SCB measure, adjusted for baseline VABS Adapt and VABS Social, was statistically significant, and the effect size approached medium size. It is noteworthy that the unadjusted large effect size presented in Table 6 is untrustworthy due to the specification error, as it does not adjust for the two covariates that, together, accounted for 15% of the variation in ΔSCB (*R*^*2*^ = .15, *f*(2,53) = 4.599, *p* = .014). Finally, adjusting for age, baseline MSEL, and VABS Adapt, the Pathways group made significantly more change in VABS Adapt, and the magnitude of the effect was considered medium, as was the unadjusted effect size seen in Table 6. Together, the covariates accounted for 34% of the variation in the change in adaptive functioning (*R*^*2*^ = .34, *f*(3,52) = 8.90, *p* = .0001). No significant interaction effects were found when comparing the Pathways and SAU groups.

### Pathways and Communication Group Comparison

The regression coefficients on the dummy-coded group variable and the associated partial *F*-statistic and effect size estimates for the outcome variables are presented in Table 9. Any covariates that were entered into the model before the group variable are listed in the first column of the table. When covariates were entered in the model, as they are for ΔSocial Eye Gaze, ΔComm, and ΔVABS Adapt, the regression coefficients reflect the adjusted mean difference in change on the outcome measures. The results suggest that, adjusting for baseline MSEL, the Pathways group made statistically more change on social eye gaze compared to the communication group, and the effect was of medium size.

**Table 9:** Unstandardized regression coefficients and confidence intervals for the dummy-coded group variable, adjusted for covariates, and the associated partial F-statistic and effect size estimates for the comparison of Pathways and communication intervention

Covariate	*b*_1_	95% CI for *b*_1_		*SE*	*Partial F*	*df*	*p* value	R^2^/ΔR^2^	*F*^2^
		LL	UL						
			ΔSocial Eye Gaze						
MSEL	15.80	6.18	25.41	4.79	10.88	1, 51	.002	.16	.19
			ΔVABS social						
None	6.66	1.14	12.19	2.75	5.85	1, 52	.019	.10	.11
			ΔComm						
VABS adapt	− 0.02	0.29	− 0.01	.288	0.003	1, 51	.956	.00	.00
			ΔSCB						
None	1.42	− 4.70	7.55	3.05	0.22	1, 52	.643	.04	.001
			ΔVABS adapt						
MSEL	15.0	2.58	27.82	6.29	5.85	1, 51	.019	.09	.10

*f*^2^ = effect size, where ≥.02 = small effect, ≥.15 = medium effect, and ≥.35 = large effect

*b*, unstandardized beta, CI, confidence interval around the unstandardized beta; LL, lower limit of confidence interval; UL, upper limit of confidence interval; MSEL, Mullen Scales of Early Learning, Early Learning Composite score; VABS Social, sum of raw scores of the Vineland Adaptive Behavior Scale, Second Edition Socialization domain score; Comm, a communication measure that composites the number and diversity of communicative acts; VABS adapt, sum of raw scores of the Vineland Adaptive Behavior

The unadjusted large effect presented in Table 6 is untrustworthy due to the specification error, as it does not account for MSEL. Alone, MSEL accounted for 8% of the variation in ΔSocial Eye Gaze (*R*^*2*^ = .08 *f*(1,52) = 4.52, *p* = .038). The Pathways group also made significantly more change on VABS Social, and the magnitude of the effect approached medium size, which is consistent with the effect size reported in Table 6. There were no significant differences between the Pathways group and the communication group on the two communication measures. Interestingly, the results in Table 6 suggests that there may be a small effect of Pathways on change in SCB; however, our study was not powered to find small effects. Finally, adjusting for baseline MSEL, the Pathways group made significantly more change on VABS Adapt, and the effect was considered small to medium. The covariate, baseline MSEL, accounted for 10% of the variation in ΔVABS Adapt (*R*^*2*^ = .10, *f*(1,52) = 5.95, *p* = .018). No significant interaction effects were found when comparing the mutual-gaze and communication groups.

## Summary and Discussion

The current study examined the efficacy of Pathways, a parent-mediated NDBI for early autism that fits the guiding principles and service delivery model of IDEA Part C programs for which the number of weekly visits by an interventionist may be constrained due to limited resources. Families were visited in their homes once a week for 1.5 h. The intervention coached parents to facilitate early-developing dyadic social skills through a series of systematic instructional strategies that move parents toward increasingly more sophisticated interactions with their toddler. Of particular interest for this research was the instructional strategy that coached parents to actively engage their toddler in mutual gaze during motivating face-to-face routines, followed by contingent natural reinforcement. Mutual gaze is an important component of early dyadic interaction in typical infants that may activate the social brain network (Johnson et al. 2015; Jones and Klin 2013; Senju and Johnson 2009). We hypothesized that engaging toddlers with ASD in dyadic face-to-face reciprocal interactions with mutual gaze may be pivotal for social development, and would have positive effects on other areas of development.

A total of 78 culturally and economically diverse families with ECI-aged toddlers completed the study. The family demographics and cognitive level of the toddlers were roughly equivalent to statewide ECI demographics (Texas Health and Human Services n.d.), suggesting that the families in the study were representative of the state’s ECI population. Participating families were randomly assigned to one of three 12-week interventions, i.e., Pathways, communication, and SAU who received services from community providers. The Pathways and the communication groups were both project-related, parent-mediated NDBIs that focused on dyadic face-to-face reciprocal interactions, animation, and imitation within social sensory and daily routines. The Pathways and communication groups differed in that the former embedded an instructional strategy for mutual gaze, whereas the latter embedded an instructional strategy to facilitate communication. The Pathways/SAU group comparison concerned the efficacy of the Pathways intervention as a potential ECI intervention; the Pathways/communication group comparison concerned the efficacy of facilitating mutual gaze—rather than social communication—in dyadic social interactions as an active ingredient for social development.

Our findings support the Pathways intervention as a potential intervention for toddlers with ASD enrolled in IDEA Part C programs. Specifically, as compared to the SAU group, the Pathways group made significantly more progress on the two social measures (ΔSocial Eye Gaze and ΔVABS Social) and on adaptive functioning. The effects of the Pathways intervention on change in adaptive functioning was adjusted for age, baseline development, and adaptive functioning that, together, accounted for an additional 34% of the variation in the change in adaptive functioning. Importantly, when compared to SAU, the effect of the Pathways intervention on our two measures of social skills and on adaptive functioning was large.

There was no significant difference between the Pathways and SAU groups on change in communication, a composite measure of the total number of communicative acts, and the diversity of communicative acts. After adjusting for baseline VABS Social and adaptive functioning, however, the Pathways intervention had a small to medium effect on synchrony of communicative behaviors, which concerned the social sophistication of communicative behaviors. For all groups, progress in the communication measure was realized predominantly by increasing the number of behavioral regulations. Behavioral regulations are instrumental intentions used to regulate or influence the behavior of others. They develop early and are less socially sophisticated than are intentions used to share attention (Camaioni 1993; Tomasello et al. 2005). Taken together, the findings for the communication measures suggest that the SAU group made little change in communication with their parents, and the sociability of their communication was not well developed. In contrast, the Pathways group used similar rudimentary intentions but produced them using more socially sophisticated communicative behaviors, such as combining words/vocalizations with eye contact or gestures.

It is noteworthy that toddlers in the SAU group received more hours of services, on average, than did the children in the Pathways group, who received 1.5 h/week. This is consistent with Fuller and Kaiser (2019) meta-analysis that found that dosage, defined by number of intervention hours, was not a significant predictor of treatment effect. Fewer professional hours may be needed when interventionists coach and support family members on how to engage the toddler with ASD on developmentally accessible social skills during meaningful family activities and daily routines appropriate to the family’s culture (Schertz et al. 2018; Siller et al. 2013; Wetherby et al. 2018).

We should emphasize that there are several features of the Pathways intervention that may have contributed to its effects as a potential ECI program. First, Pathways is a developmentally informed, targeted intervention that focuses on the period of shared emotions, which is developmentally earlier than shared attention (i.e., joint attention) and communicative intention (Adamson and Russell 1999; Tomasello et al. 2005). Second, parents were coached using adult learning strategies that foster active learning, such as in vivo feedback/guidance, self-reflection, demonstration, and engagement with material within an authentic experience, that have a research base (Dunst, and Trivette 2012; Friedman et al. 2012; Rush and Shelden 2011) and are recommended by the DEC (2014). Third, many of the interactional strategies (i.e., limiting distractions and demands, wait time, face-to-face positioning, contingent natural reinforcement, and embedding mutual gaze, animation and imitation in child-specific dyadic face-to-face social sensory and family routines) are strategies that have been used in intervention research on young children with ASD (Brian et al. 2017; Ingersoll and Gergans 2007; Kasari et al. 2015; Koegel et al. 2009; Schertz et al. 2013; Wallace and Rogers 2010, Wetherby et al. 2018).

In the current study, we were interested in the effects of embedding mutual gaze into dyadic face-to-face interactions. To that end, we compared two project-related intervention groups: Pathways and communication interventions. We found that Pathways had a medium effect on changes in social skills (ΔSocial Eye Gaze and ΔVABS Social) and a small effect on changes in adaptive functioning when compared to the communication intervention. In addition, Pathways had no effect on communication measures (ΔComm and ΔSCB) when compared with the communication intervention, which is noteworthy, as the communication intervention coached parents on strategies to facilitate communication, and the Pathways intervention did not.

The magnitude of the effect of the Pathways intervention on social skills was large when compared with SAU but only medium when compared with the communication group. The differences in effect sizes between the Pathways intervention and the two comparison groups could mean that the coaching and interactional strategies that were common to the two project-related interventions played a role in facilitating social skills or that the specific coaching strategies to prompt communication improved social skills, although to a lesser degree than strategies to facilitate mutual gaze. While this study does not allow us to identify which of these interpretations is correct, the findings suggest that social skills were enhanced by mutual gaze.

Thus, the findings support our hypothesis that embedding strategies for mutual gaze within face-to-face reciprocal interactions is important to the development of social skills and has a cascading influence on communication development. In addition, the results lend support to joint attention-mediated learning (Schertz et al. 2018) and the Social ABCs (Brian et al. 2017), which are two interventions that focus on early social interactions similar to the dyadic interactions promoted in the Pathways intervention. These social skills are observed between parents and typical infants around 2 to 6 months of age during the social-cognitive phase of sharing emotions and are putative precursors to social communication (Adamson and Russell 1999; Rollins and Greenwald 2013; Tomasello et al. 2005). Both joint attention-mediated learning and the Social ABCs realized positive effects on measures of dyadic interaction, including looking at faces and social orienting. In addition, both interventions found positive effects on developing social communication skills. The implication for ECI providers is to provide families who have toddlers with ASD with the skills to promote early-developing social skills. Our findings suggest that this may be accomplished by implementing the coaching and instructional strategies described above, facilitating early social interaction skills, generally, and embedding mutual gaze into dyadic face-to-face interactions, specifically.

### Limitations

The difference between the Pathways and communication group interventions was only one unit (i.e., Unit 3). Due to the similarities between the interventions, we might have expected the effect size for the Pathways/communication group comparison to be small, as was the case for change in SCB. One limitation of this study is that it was not powered to find small effects. It is noteworthy, however, that the two project-related interventions focused on developmentally informed early social skills and that the units were cumulative. The progressive nature of the intervention allowed parent-toddler dyads to develop increasingly more sophisticated interactions before the parent added new and perhaps more difficult elements into the interaction. Therefore, although the difference between the groups was just a single unit, parents embedded the unit’s strategies into their interactions for 5 to 7 weeks.

Another potential limitation is that all of the outcome measures were obtained during a parent-toddler interaction or by parent report. Because we were evaluating a parent-mediated intervention, parents implemented the intervention and, therefore, were not blind to the intervention condition. This suggests that the Pathways and communication groups had an advantage over the SAU group, as they could utilize facilitative strategies learned during the intervention phase (Yoder and Crandall 2019). Similarly, it is not known whether the results found for parents would generalize if unfamiliar adults were interacting with the toddlers (Yoder and Crandall 2019). Future research should include standardized assessments, such as the Communication and Symbolic Behavior Scales, Developmental Profile (Wetherby and Prizant 2002), implemented by unfamiliar interventionists to address the consistency of measurements across groups and generalizability.

Another limitation was the short duration of the 12-week intervention, coupled with the lack of a follow-up assessment several months after families finished the study. It may be that 12 weeks is too short a time for parents to maintain the interactional strategies learned in the study, especially if they receive conflicting messages from professionals when returning to SAU programs. The results need to be replicated and include follow-up assessment 3 and 6 months post-intervention to determine whether the effects are sustained. Finally, although interventionists were blind to group assignment at intake, a limitation of this study is that interventionists were not blinded when administering post-intervention assessments. Fuller and Kaiser (2019) found the effects from this risk to be low; nonetheless, to reduce bias, an interventionist who was not familiar with the family conducted their assessments.

## Conclusions

Despite these limitations, this study illustrates the efficacy of the Pathways intervention in a group of culturally and socioeconomically diverse families that could be implemented in IDEA Part C programs. Further, the toddlers in the Pathways intervention, in which parents were coached to use strategies to embed mutual eye gaze in face-to-face reciprocal interactions, made significantly more changes in social skills when compared with the communication control, and the magnitude of the effect was medium. In contrast, the Pathways and communication groups performed similarly on changes in communication skills. These findings suggest that mutual eye gaze, when embedded in dyadic face-to-face reciprocal interactions, may be an active ingredient for social development, with cascading effects on changes in communication skills in cognitively impaired toddlers with ASD.

An important next step is to replicate these findings with Part C providers in the context of their hectic work schedules. Notably, there are potential challenges to community-based studies stemming from the broader, more variable population of toddlers served, which could decrease the size of the intervention effect (Nahmias et al. 2019) and necessitate a larger sample. A strength of the current study was that the family demographics and cognitive level of the toddlers were commensurate with state funded ECI programs. However, state-established eligibility criteria are based on degree of delay in one or more areas of development, and diagnosing ASD is outside the purview of Part C. This means that without imposing researcher control, many Part C toddlers with ASD will not have a confirmed diagnosis, increasing the heterogeneity of community-based samples. Systematic professional development concerning red flags for ASD, early social development and job embedded intervention training may improve providers’ accuracy and skill in identifying toddlers at risk for ASD and in implementing the intervention (Wetherby et al. 2018). In addition, the developmental nature of the Pathways intervention may benefit toddlers who are demonstrating deficits in reciprocal dyadic interactions and who have not yet acquired joint attention, even if they do not have ASD. Identifying effective, early ASD interventions that can be used in community-based programs is crucial to help states to offer cost-effective models, build capacity, and break down barriers to timely intervention.

## Footnotes

1 The term *parent* is used to refer to any primary caregiver who lives with the child.
